# Schwartz–Jampel syndrome is not related to malignant hyperthermia

**DOI:** 10.1186/s40981-017-0104-7

**Published:** 2017-06-06

**Authors:** Kohei Godai

**Affiliations:** Department of Anesthesiology and Critical Care Medicine, Graduate School of Medical and Dental Sciences, Kagoshima University, 8-35-1 Sakuragaoka, Kagoshima, 890-8520 Japan

**Keywords:** Malignant hyperthermia, Myotonia, Schwartz–Jampel syndrome

## Abstract

Schwartz–Jampel syndrome (SJS) is a rare syndrome that is clinically characterized by myotonia and skeletal abnormalities. Most reports regarding SJS have stated that patients with SJS are susceptible to malignant hyperthermia (MH). The statement is incorrect. There is no report showing that SJS is related to MH. Scientific evidence also shows that patients with myotonias are not susceptible to MH except for that with hypokalemic periodic paralysis. Medical practitioners must recognize that SJS is not related to MH.

## Correspondence

To the Editor:

I and my colleagues recently published a case report describing a successful airway management in a patient with Schwartz–Jampel syndrome (SJS) [1]. SJS is a rare syndrome that is clinically characterized by myotonia and skeletal abnormalities. Most previous reports regarding SJS have stated that patients with SJS have increased risk for malignant hyperthermia (MH) [2, 3]. After reviewing the literature, however, I found that the statement is incorrect. There is only one case report which describes intraoperative thermoregulatory dysfunction in a 23-month girl with SJS [4]. The authors reported that “her temperature rose 1.5 °C within ten minutes” after administration of atropine, ketamine, nitrous oxide, and curare. The patient’s temperature returned normal within 30 min without any medications, and the patient was discharged the following day uneventfully. I do not believe that the increased body temperature was related to MH, because the authors did not use volatile anesthetics or depolarizing muscle relaxants. I do not think that patients, who developed MH, will return to normal body temperature in a short time without being administered dantrolene. In addition, scientific evidence shows that patients with myotonias are not susceptible to MH except for that with hypokalemic periodic paralysis [5]. The authors of previous reports might be misled by the title “Malignant hyperpyrexia in a patient with Schwartz-Jampel syndrome” used in the case report [4]. It is impossible, however, to completely exclude the possibility of MH among the patients with SJS, because SJS is a rare syndrome. Anesthesiologists should administer MH triggering agents to these patients with meticulous caution. It is very important to keep dantrolene immediately accessible in the operating room.

The fear that SJS is susceptible to MH might lead medical practitioners to avoid necessary general anesthesia in patient with SJS. Necessary general anesthesia should never be avoided, since patients with SJS do not have increased risk of MH. I believe that this article provides useful information regarding SJS to medical practitioners and patients. Medical practitioners must recognize that SJS is not related to MH.

## Abbreviations

MH: Malignant hyperthermia
SJS: Schwartz–Jampel syndrome
